# The Influence of Lifestyle on Male Fertility in the Context of Insulin Resistance—Identification of Factors That Influence Semen Quality

**DOI:** 10.3390/jcm13102797

**Published:** 2024-05-09

**Authors:** Adrianna Zańko, Iwo Martynowicz, Anna Citko, Paulina Konopka, Adam Paszko, Michał Pawłowski, Łukasz Szczerbiński, Katarzyna Siewko, Adam Jacek Krętowski, Waldemar Kuczyński, Robert Milewski

**Affiliations:** 1 Doctoral School, Medical University of Bialystok, 15-276 Bialystok, Poland; adrianna.zanko@sd.umb.edu.pl; 2 Center for Reproductive Medicine KRIOBANK, 15-879 Bialystok, Poland; iwo.martynowicz@gmail.com (I.M.); kuczynski.kriobank@gmail.com (W.K.); 3 Clinical Research Center, Medical University of Bialystok, 15-276 Bialystok, Poland; anna.citko@umb.edu.pl (A.C.); paulina.konopka@umb.edu.pl (P.K.); adam.paszko@umb.edu.pl (A.P.); 4 Department of Biostatistics and Medical Informatics, Medical University of Bialystok, 15-295 Bialystok, Poland; michal.pawlowski@umb.edu.pl; 5 Department of Endocrinology, Diabetology and Internal Medicine, Medical University of Bialystok, 15-276 Bialystok, Poland; lukasz.szczerbinski@umb.edu.pl (Ł.S.); katarzyna.siewko@umb.edu.pl (K.S.); adam.kretowski@umb.edu.pl (A.J.K.)

**Keywords:** male fertility, insulin resistance, sperm motility, lifestyle

## Abstract

**Background**: Male fertility is known to have been negatively influenced by the progress of civilization. Another condition whose incidence has been on the increase for the same reason is insulin resistance (IR). In addition, men increasingly often resign from the pursuit of active forms of leisure, preferring more sedentary ones. Considering these trends, this aim of this study was to investigate the relationships between lifestyle factors, insulin resistance, and male fertility in men with and without the condition. A further aim was to select those lifestyle factors that would make it possible to predict the level of male fertility, especially when IR is concerned. **Methods**: This study was performed in a group of 73 participants, divided into groups based on their insulin resistance status. Their physical activity, diet, perceived stress, sleep quality, libido level, and duration of sexual abstinence were assessed on the basis of a number of parameters, including indices proposed by the authors. In addition, relevant anthropometric measurements were taken and tests related to glucose metabolism and semen quality were carried out. On the basis of these data, statistical tests were performed to establish or disprove relationships between lifestyle choices and semen quality, as measured my sperm motility. **Results**: The results of this study highlighted the associations between a number of parameters, i.e., micronutrient and vitamin intake, diet quality, body composition, insulin resistance, and the duration of sexual abstinence, and semen quality, as measured by sperm motility. Significantly, the presence or absence of IR was linked to male fertility. A multivariate model was developed, incorporating parameters such as the Matsuda index, vitamin intake, and sexual abstinence duration, to predict motility scores. **Conclusions**: This study underscores the negative impact of modern civilization’s lifestyle choices on male fertility. Notably, vitamin and mineral consumption, especially from antioxidant-rich diets like the Mediterranean diet, emerged as key modifiable factors affecting fertility. Routine diagnostics for insulin resistance in fertility-related interventions is recommended. This study also highlights the importance of considering sexual abstinence duration during semen collection for accurate diagnostic results. Future research should focus on validating the proposed multivariate model and exploring the effects of lifestyle modifications, particularly vitamin supplementation, on fertility outcomes in men, especially in the context of IR.

## 1. Introduction

Infertility is defined by the World Health Organization (WHO) as a disease of the male or the female reproductive system characterized by the failure to achieve a pregnancy after 12 months or more of regular unprotected sexual intercourse [1]. Globally, it affects approximately 70 million people and it is estimated that 50% of all infertility cases result from problems with the male reproductive system. Numerous factors, such as genetic mutations or comorbidities, influence male fertility, with studies also pointing to lifestyle choices as a significant contributing factor [1].

Sperm quality analysis is regarded as the basic method of male fertility assessment [2]. It consists of quantitative and qualitative measurements in relation to reference ranges [3]. According to the latest standards, i.e., the sixth edition of the WHO Manual for the Laboratory Examination and Processing of Human Semen [4], semen volume should be at least 1.4 mL while the optimal total sperm number should be 39 × 10^6^ per ejaculate. The reference values of the other parameters are expressed in percentages, i.e., the optimal total motility should be 40% and progressive motility should be 32%, while normal forms (also known as morphology) should be 4% [4,5]. In terms of lifestyle, the aforementioned parameters are influenced by a number of factors such as BMI (Body Mass Index), diet, physical activity, sleep, using drugs—including alcohol or cigarettes—or sexual abstinence. External factors such as environmental pollution, elevated temperature in the testicular area, or radiation also influence semen quality [6].

Studies on male fertility often address the issue of obesity and its impact on fertility. The primary processes identified as disrupting erectile function in obese individuals include ongoing inflammation within the body [7], leptin resistance [8], insulin resistance [9,10], and oxidative stress [11]. Additionally, ejaculatory dysfunction may lead to low semen volume and reduced sperm count, hindering conception [12].

Insulin resistance is a condition in which the insulin circulating in the blood is unable to properly stimulate glucose uptake and/or use by insulin-sensitive organs and tissues. In a healthy body, pancreatic β-cells respond to increased blood glucose levels by producing insulin and inhibiting glucose production in the liver. In insulin resistance, not all signals are read correctly and thus the body increases the production both of insulin in the pancreas and glucose in the liver [13].

The regulation of glucose levels is not the only function performed by insulin in the body. The compound also plays an important role in the regulation of lipid metabolism. In the case of insulin resistance, increased lipogenesis in the liver is observed. This process ultimately results in non-alcoholic fatty liver disease involving the accumulation of fat in the liver [13]. The fatty growth of internal organs and the subsequent β-cell hyperplasia lead to abnormal insulin secretion, exacerbating insulin resistance, with the process repeating in a circular pattern [14].

The HOMA-IR (Homeostatic Model Assessment of Insulin Resistance) is the most frequently used index for measuring insulin resistance. It is calculated on the basis of fasting insulin and glucose levels and interpreted in the following manner: if the score is >1, it is considered abnormal; if the score is >2, it indicates insulin resistance [15]. Another indicator that is widely used to determine the opposite relationship, i.e., tissue insulin sensitivity, is the Matsuda index, calculated on the basis of plasma glucose concentration and insulin concentration obtained from fasting blood samples after oral consumption of 75 g of glucose during the OGTT, i.e., the oral glucose tolerance test [16].

Although insulin resistance is more commonly diagnosed in women, some studies, in fact, suggest that men have lower peripheral insulin sensitivity than women [17,18]. This may result from differences in the levels of sex hormones in the blood or from the distribution of adipose tissue, which, as far as insulin resistance is concerned, is less favorable in men. The results of a study performed by Li et al. demonstrated a greater insulin resistance of adipose tissue in men compared to women with similar BMI levels. Additionally, testosterone levels were inversely correlated with the level of insulin resistance of adipose tissue. This means that, among men, the higher the testosterone level, the lower the level of insulin resistance; among women, this relationship was reversed [19]. Some studies also suggest a protective role of estrogens in the context of insulin resistance; however, most of them have so far been conducted only on animal models [20].

Insulin resistance in men is often associated with obesity and metabolic syndrome. These conditions are connected with a deterioration in fertility, in addition to decreased levels of general health. The impact of insulin resistance on male fertility specifically has not been widely studied. Nevertheless, indirect associations can be found, suggesting that it affects the quality of semen and the levels of sex hormones in men [6]. Among the studied relationships between male fertility and semen quality, the impact of insulin resistance on the concentration and production of testosterone [21,22,23], the reduction in semen volume, or the percentage of progressive sperm [21] can be mentioned, among others.

An indirect relationship between insulin resistance and sperm quality can be further linked to lifestyle. This is due to the fact that many of its components influence both insulin resistance and male fertility. These include diet, physical activity, sleep, stress, environmental pollution, and the duration of sexual abstinence [6,24,25,26,27,28]. Generally speaking, sperm quality is lower nowadays compared to past decades, which indicates that the problem can be linked to various civilization factors [6], thus making it necessary to study their influence on fertility.

Due to the fact that motility is a very important parameter in terms of semen quality [1] and considering its association with insulin resistance, it was adopted in this study as the indictor of male fertility. Suboptimal semen quality outcomes are frequently connected with the presence of metabolic syndrome, characterized by elevated BMI levels and the presence of metabolic dysfunctions, including dyslipidemia and insulin resistance [29]. Moreover, untreated insulin resistance can lead to hyperglycemia, which increases the production of advanced glycation end products, associated with the generation of reactive oxygen species, also within the testes and epididymis, damaging sperm DNA [30]. It has also been reported that the use of metformin, i.e., a drug used both in diabetes and in insulin resistance without the presence of diabetes, improved sperm count, morphology, and motility [31]. The aforementioned global trends consisting of progressively worsening numbers and healthy sperm motility in the ejaculate are associated with an increased risk of infertility [1]. Studies show that total progressively motile sperm count (TPMSC) may be used as a predictor of positive pregnancy test results in non-donor IUI cycles. It is worth noting that a decreased level of initial sperm motility in the process of semen preparation used in IUI—which significantly improves sperm motility—lowers the probability of a positive pregnancy outcome [32,33]. In terms of the relationship between sperm motility and insulin resistance, it needs to be emphasized that glucose metabolism plays an important role in spermatogenesis. Basic cell activities and their specific characteristics, such as motility and the activity of a mature sperm that leads to fertilization, are maintained by glucose metabolism, with insulin resistance disturbing the process [34].

A modifiable element of the modern lifestyle that has been changing in previous decades is diet. The popular Western-style diet, low in vitamins and high in highly processed products, has been shown to pose numerous health risks [35]. The Mediterranean and the DASH (Dietary Approaches to Stop Hypertension) diets, on the other hand, appear to be the most effective ones in terms of the treatment and prevention of various conditions, including insulin resistance. Both are based on antioxidant ingredients, i.e., vitamins and minerals, which reduce inflammation in the body [35,36,37]. It has been proven that the diets in question have a positive effect on semen quality, improving parameters such as the total sperm count, sperm concentration, and the number of progressive sperms [38,39,40].

Another dietary factor that has an impact on male fertility are vitamins. Regardless of whether men adhere to a special diet or not, antioxidant vitamins appear to be extremely important as far as the improvement in semen parameters is concerned, motility in particular. Vitamin E acts as an antioxidant that breaks the chains of peroxide radicals, while vitamin C prevents lipid peroxidation, thus reducing the amount of free oxygen radicals [39]. The rationale of using vitamins in the treatment of male infertility has been supported in animal studies. A 2020 study performed in mice showed that supplementation consisting of combined ascorbic acid and tocopherols (60% γ-tocopherol) could restore fertility induced by oxidative stress. Male mice fed a supplemented diet showed improvement in semen quality, including motility and progressive motility [41]. Insulin resistance can be induced by oxidative stress via the interference with insulin signal transmission and deregulation of adipokines, as well as inflammation—occurring in the course of both insulin resistance and oxidative stress [42].

As far as other lifestyle-related factors are concerned, research focused on moderate physical activity confirms its positive effect on both tissue insulin sensitivity [43] and the improvement in semen quality [28]. However, high-intensity physical activity may result in abnormalities. A meta-analysis performed by Ibañez-Perez et al. showed that too intense a training may lead to a decreased progressive movement of sperm, as well as reduced parameters such as sperm concentration, volume, and morphology [44]. Stress is another important factor in the context of male fertility. Physiological stress triggers a cascade of free radicals and pro-inflammatory cytokines that have the ability to damage sperm. It is also a factor that plays a role in insulin resistance as the condition is intensified by stress [45].

As far as diagnostic aspects are concerned, research on semen quality shows that the duration of sexual abstinence needs to be taken into consideration in order to obtain reliable fertility results. For this reason, it should range from approximately 2 to 7 days prior to the test [46]. The most beneficial duration of sexual abstinence varies for individual semen parameters, with some of them improving and others weakening depending on their duration. Sperm motility, morphology, and the percentage of DNA fragmentation tend to improve during a shorter period of abstinence, i.e., lasting up to 4 days, while semen volume and the total sperm count reach the highest values after the 5th day of sexual abstinence, increasing in a directly proportional manner [47,48].

Due to the considerable number of literature reports linking the factors described above with male fertility, the aim of this study was to experimentally confirm or disprove the relationship between lifestyle factors such as physical activity, diet, perceived stress, sleep quality, and the duration of sexual abstinence with semen quality in the context of insulin resistance. A further aim was to select those lifestyle factors that would make it possible to predict the level of male fertility, especially when insulin resistance is concerned.

## 2. Materials and Methods

The clinical–control study was conducted in 73 men with and without fertility impairment, divided according to the presence or absence of insulin resistance. The participants were patients of the KRIOBANK Infertility Treatment Clinic and the Department of Endocrinology, Diabetology and Internal Medicine of the Medical University of Bialystok. All participants were obliged to complete their personal 3-day food diaries. They were instructed by the researchers that the collected data must reflect the dietary habits of the participants, as opposed to providing information on the three days immediately before semen collection, which for various reasons could be atypical. Moreover, to ensure accuracy, a dedicated researcher was present during the collection of the data and verified their reliability. Hence, the three-day recall reflected three typical days preceding semen collection.

The following inclusion criteria were used: age >18 years, presence (study group) or absence (control group) of insulin resistance, and absence of treatment for chronic diseases. The exclusion criteria were the presence of a diagnosis of infertility or diabetes (both in the study and control groups). All participants gave their consent to participate in this study by signing the appropriate Informed Consent forms.

The presence of insulin resistance was established in the following manner:

(a) HOMA-IR was calculated as follows: fasting insulin (μIU/mL) × fasting glucose (mmol/mL)/22.5;
(b) Matsuda Index was calculated as follows: 10,000/√[fasting glucose (mmol/L) × fasting insulin (pmol/L)] × [mean glucose (mmol/L) × mean insulin (pmol/L) during OGTT] [49].

After the above indices were established, the cut-off point for insulin resistance was established in the following manner:

(a) NOMA-IR index: R = fasting insulinemia (mU/mL) × fasting glycemia (mmol/l)/22.5; R > 0.91 ± 0.38 indicated the presence of insulin resistance;
(b) Matsuda index: 100,000/fasting insulinemia (mU/mL) × fasting glycemia (mg/dl) × mean glycaemia value during OGTT × mean insulinemia value during OGTT; Matsuda index values < 7.3 may indicate the presence of insulin resistance [50].

Table 1 presents the two cohorts of subjects divided into those with and without insulin resistance (according to Matsuda index scores) together with their characteristics.

This study focused on the following lifestyle factors: physical activity, diet, perceived stress, sleep quality, libido level, and the duration of sexual abstinence. Additionally, in order to make it possible to quantitatively assess and compare the quality of the participant’s lifestyles with other parameters, original indices were created based on the recommendations proposed by the World Health Organization and the Polish Institute of Food and Nutrition [48]. This solution made it possible to determine the subjects’ adherence to healthy eating habits and the adequate level of physical activity for healthy men. The following indices were used:

(1) Vitamin Consumption Index (VCI) (0–10 pts)—each vitamin consumed in amounts specified in the recommendations was awarded 1 pt. The following ten vitamins were considered in the analysis: vitamin E, vitamin A, thiamine, riboflavin, niacin, vitamin B6, vitamin C, folic acid, vitamin D, and vitamin B12.
(2) Mineral Consumption Index (MCI) (0–9 pts)—each mineral consumed in amounts specified in the recommendations was awarded 1 pt. The following nine minerals were considered in the analysis: sodium, potassium, magnesium, phosphorus, calcium, iron, zinc, copper, and manganese.
(3) Macronutrient Consumption Index (MacCI) (0–5 pts)—each of the four macronutrients consumed in amounts specified in the recommendations was awarded 1 pt.; 1 pt. was also awarded for adequate caloric intake. The following five parameters were considered in the analysis: fat, carbohydrate, fiber, dietary fiber, and caloric intake.
(4) Dietary Index (DI) (0–24 pts)—this index is calculated as VCI + MCI + MacCI.
(5) Body Composition Index (BCI) (0–3 pts)—each instance of a body composition norm met by the participant was awarded 1 pt. The following three parameters related to body composition were used to calculate the value of the BCI index: Total Body Water (TBW), Body Fat Mass (BFM), and Skeletal Muscle Index (SMI).

The indices described above were established on the basis of data on the consumption of vitamins, minerals, calories, and macronutrients obtained from the 3-day food diary (dietary recall), and then analyzed in the Dieta 6 software from the National Institute of Public Health (Warsaw, Poland). Then, the data were compared to the dietary norms for the general Polish population, taking into account the EAR (Estimate Average Requirement) level [51]. BCI was determined by performing body composition analyses and comparing the results with the applicable norms [51].

Furthermore, anthropometric measurements of height, body weight, and waist and hip circumferences were taken. In addition, tests of fasting glucose and insulin concentrations at 30, 60, 90, and 120 min of the oral glucose tolerance test were performed. Based on the glycemia and insulinemia values, the following indicators of insulin resistance were calculated: the HOMA1-IR and the Matsuda index (insulin sensitivity).

Semen quality tests were performed and analyzed using the CASA system (SCA Sperm Class Analyzer 6.6.0.6 from Microptic, Barcelona, Spain), with the following five basic parameters taken into account: volume per milliliter, volume per ejaculate, morphology, motility, and progressive motility. Due to its association with carbohydrate metabolism and because incorrect values of the parameter signify problems with fertility, special attention was paid to motility. Table 2 summarizes the results for different motility parameters in the two cohorts of subjects included in this study (with and without insulin resistance).

Due to the lack of normality of the distribution of the analyzed numerical parameters, non-parametric tests were used. The Spearman’s rank-order correlation coefficient was also determined in order to determine the relationship between two numerical or ordinal variables. The non-parametric Mann–Whitney U test was used for comparisons between two independent groups. Pearson’s Chi2 test was used to test the relationships between two nominal variables. Univariate and multivariate logistic regression analyses were conducted for the dependent variable describing correct sperm motility. Results at *p* < 0.05 were considered statistically significant. The results were prepared in Statistica 13.3 (TIBCO Software, Palo Alto, CA. USA) and Stata 18.0 (StataCorp, College Station, TX, USA) software.

## 3. Results

### 3.1. Diet

**Figure 1:**
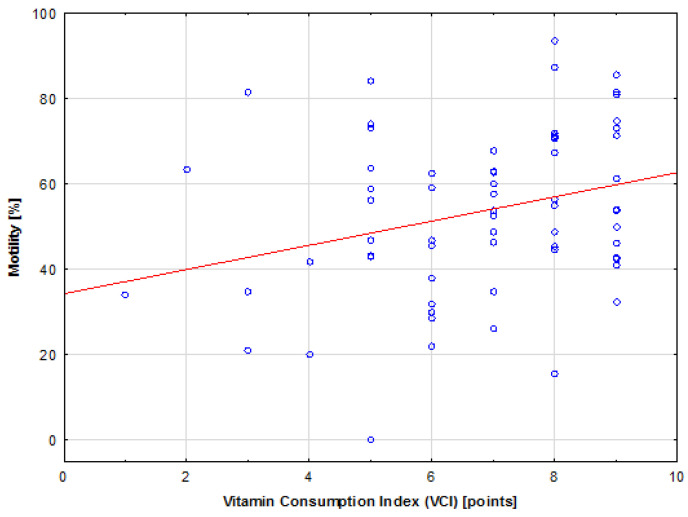
Comparison between VCI and sperm motility. The red line represents the trend line.

When comparing the parameters connected with lifestyle and those describing semen quality, it was noted that the participants characterized by greater micronutrient intake had better motility scores. In the case of vitamins, a statistically significant (*p* = 0.02), weak (R = 0.27), positive correlation was found between VCI and sperm motility. The tested relationship is presented on the scatter plot shown in Figure 1.

**Figure 2:**
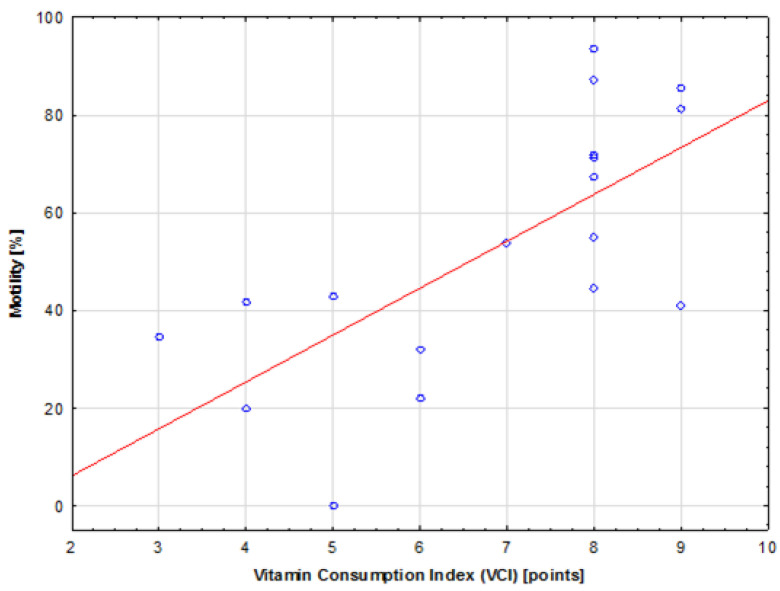
Comparison between VCI and sperm motility in the sub-group of obese participants. The red line represents the trend line.

When the aforementioned relationship was examined in sub-groups, stratified by BMI scores, a statistically significant (*p* = 0.001), strong (R = 0.70), positive correlation was found between VCI and motility for the participants in the obese group. The tested relationship is presented on the scatter plot shown in Figure 2. Conversely, no statistical significance was observed for the same relationship in the other BMI groups.

**Figure 3:**
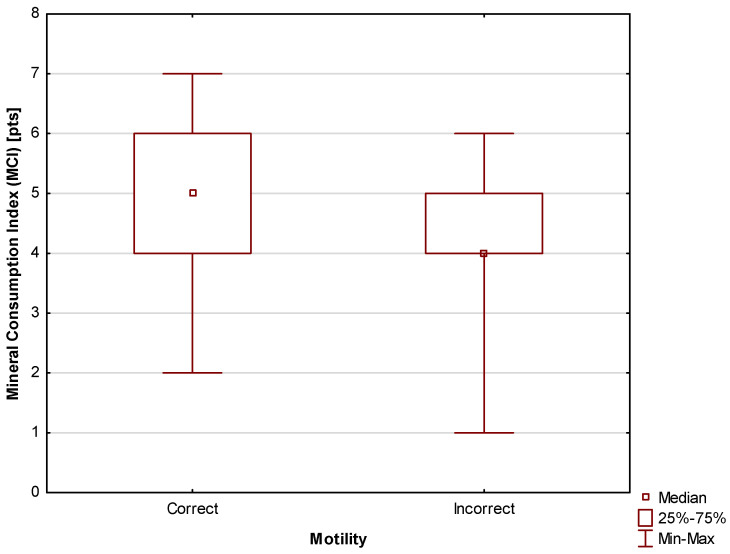
Comparison of mineral intake (MCI) between men with correct and incorrect motility scores.

Similarly to the case of vitamin consumption, statistically significant (*p* = 0.02) differences in the consumption of minerals (MCI) were found between participants with correct and incorrect motility scores. The median was Me = 5 pts (Q_1_ = 4 pts; Q_3_ = 6 pts) for men with correct motility scores and Me = 4 pts (Q_1_ = 4 pts; Q_3_ = 5 pts) for men with incorrect motility scores. The differences are shown in Figure 3.

**Figure 4:**
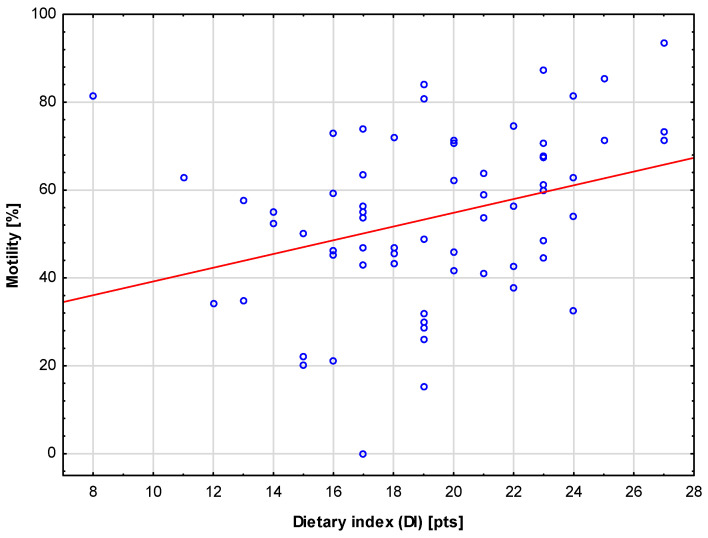
Relationship between diet quality (DI) and sperm motility. The red line represents the trend line.

Diet quality (DI) is an aggregate parameter that, in addition to VCI and MCI, reflects adequate macronutrient/caloric intake. In line with the scores obtained for VCI and MCI, a statistically significant (*p* = 0.004), average (R = 0.35), positive correlation was found between DI and sperm motility. This further corroborates the role of vitamin and mineral intake in the context of motility. The tested relationship is presented in Figure 4.

### 3.2. Body Composition

**Figure 5:**
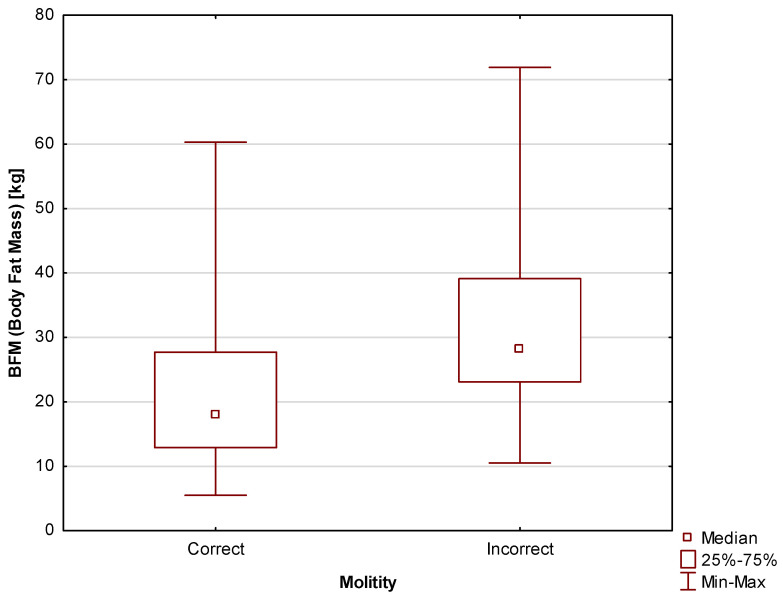
Comparison of Body Fat Mass (BFM) between men with correct and incorrect motility scores.

In terms of body composition, there were correlations between two important parameters, i.e., BFM and BCI, and motility. This indicates better scores in men with a healthier body composition. BFM differed between participants with correct and incorrect motility scores at a level of statistical significance of *p* = 0.006. The median for the study participants with correct motility scores was Me = 17.9 kg (Q_1_ = 12.9 kg; Q_3_ = 27.7 kg); for those with incorrect motility scores, the median was Me = 28.2 kg (Q_1_ = 23.1 kg; Q_3_ = 39.1 kg). The differences are shown in Figure 5.

Similarly to the case of BFM, a statistically significant (*p* = 0.04) relationship was found between body composition (BCI) and motility. A clear tendency for motility to improve together with increasing BCI was observed. At BCI = 0 pts, participants with correct and incorrect sperm motility each comprised 50% of the total number. At BCI = 1 pt., the proportion of participants with correct motility increased to 75%. Among those men for whom almost all of the body composition parameters were correct (BCI = 2 pts), participants with correct motility comprised 85.71%. As far as the participants whose body composition was completely healthy are concerned, the proportion of those with correct motility was 91.67%. The results are presented in Table 3.

### 3.3. Insulin Resistance

The results presented above, which consistently show a relationship between sperm motility and the values of indices connected with the intake of micronutrients and body composition, are further expanded with the results related to insulin resistance.

**Figure 6:**
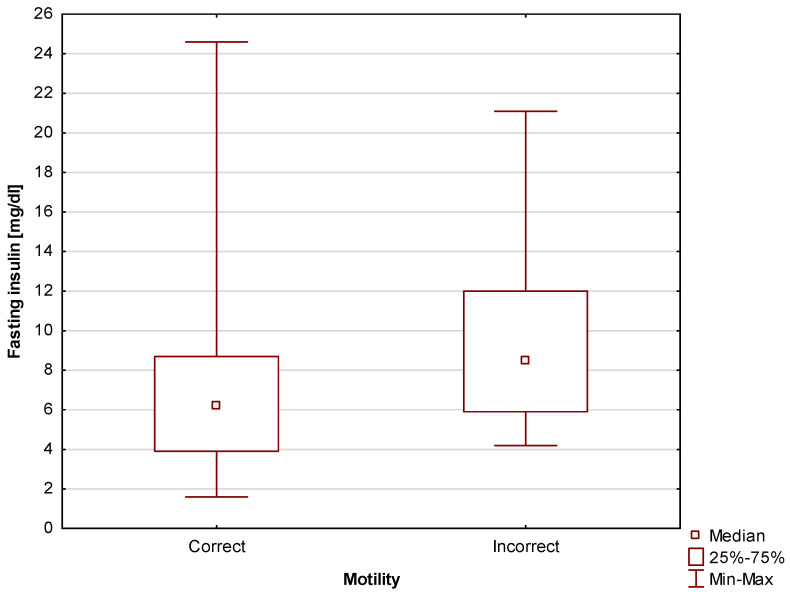
Comparison of fasting insulin level between men with correct and incorrect motility scores.

First, a statistically significant (*p* = 0.03) difference in fasting insulin level was found between men with correct and incorrect motility scores. The median was Me = 6.2 mg/dL (Q_1_ = 3.9 mg/dL; Q_3_ = 8.7 mg/dL) for men with correct motility scores, while for those with incorrect motility scores, the median was Me = 8.5 mg/dL (Q_1_ = 5.9 mg/dL; Q_3_ = 12 mg/dL). Due to the fact that this study also involved calculating the HOMA-IR index, fasting insulin level can be seen as an intermediate parameter. Nevertheless, the fact that taken in isolation, this basic parameter also shows that the relationship with motility scores is worth emphasizing. The differences are shown in Figure 6.

As could already be suspected from the fasting insulin level scores, a statistically significant (*p* = 0.04) relationship was found between the presence of insulin resistance (measured using the HOMA-IR index) and sperm motility. Correct motility scores were observed in the vast majority (82.98%) of non-insulin-resistant participants, while in the group of insulin-resistant participants, correct motility was observed in only 61.54%. These results indicate a clear connection between these two parameters. The results are shown in Table 4.

The relationship between insulin resistance and motility discussed above is further corroborated by Matsuda index scores. A statistically significant (*p* = 0.03) relationship was found between insulin resistance measured using the Matsuda index and sperm motility. Among non-insulin-resistant participants, those with correct motility scores comprised as much as 89.29% of the total number of participants. Among those diagnosed with insulin resistance, correct motility scores were observed in only 66.67% of patients. The results of tests are shown in Table 5.

**Figure 7:**
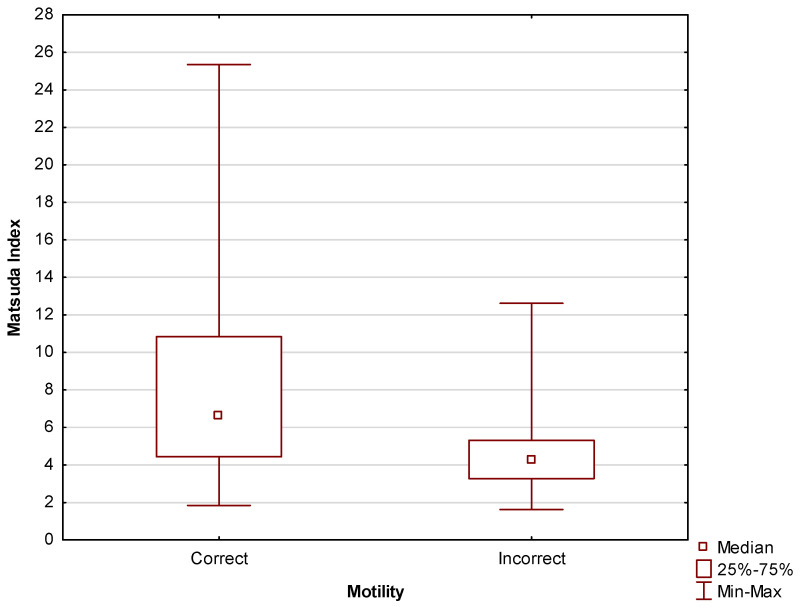
Comparison between Matsuda index scores between men with correct and incorrect motility scores.

Figure 7 shows the same relationship, but with the Matsuda index values expressed numerically. Statistically significant (*p* = 0.004) differences in Matsuda index values were found between men with correct and incorrect motility scores. The median was Me = 6.6 (Q_1_ = 4.4; Q_3_ = 10.8) for men with correct motility scores; for men with incorrect motility scores, the median was Me = 4.2 (Q_1_ = 3.3; Q_3_ = 5.3).

No statistically significant (*p* = 0.11; R = 0.19) correlation was found between the Matsuda index and sperm motility. The obtained level of statistical significance, however, combined with the low number of participants and the results of the multivariate analyses performed in this study, do suggest the possibility of the existence of such a correlation.

Due to the fact that the Matsuda index is based on insulin sensitivity, it is a more sensitive index than HOMA-IR. This is reflected in the greater number of patients who are diagnosed with insulin resistance when the former is used.

The results presented above clearly show the existence of a relationship between infertility, determined based on sperm motility; insulin resistance, measured using HOMA-IR and Matsuda indices; and dietary indices. The results show that worse motility scores were observed in men with micronutrient deficiencies and unhealthy body composition, associated with the presence of insulin resistance.

### 3.4. Sexual Abstinence

Sexual abstinence is another analyzed lifestyle-related factor, whose importance is diagnostic rather than connected with modifiability, although its analyses are informative as far as intercourse planning is concerned. Among the participants, the duration of sexual abstinence and sperm motility were found to be related at a level of statistical significance of *p* = 0.006. Among those who declared a duration of sexual abstinence of up to 4 days, correct motility was observed in 83.33%. In those who declared a duration of sexual abstinence of 4 days or more, correct and incorrect motility was reported in 50% each. The results are shown in Table 6.

In the performed univariate logistic regression analysis, a statistically significant influence on sperm motility was found in the case of the following parameters: Matsuda index, HOMA-IR index, MCI, VCI, and the duration of sexual abstinence. On the other hand, Macronutrient Consumption Index (MacCI), tobacco smoking, physical activity, BMI, and age were not found to be statistically significant. The results are shown in Table 7.

On the basis of the results of the univariate logistic regression analysis, the construction of a multivariate model was attempted. Despite the fact that a statistically significant influence on sperm motility was found in the case of some of the parameters that was found in the univariate analysis, they were not included in any of the attempted models. Eventually, a model based on the following three parameters was proposed, i.e., Matsuda index, VCI, and the duration of sexual abstinence (Table 8).

**Figure 8:**
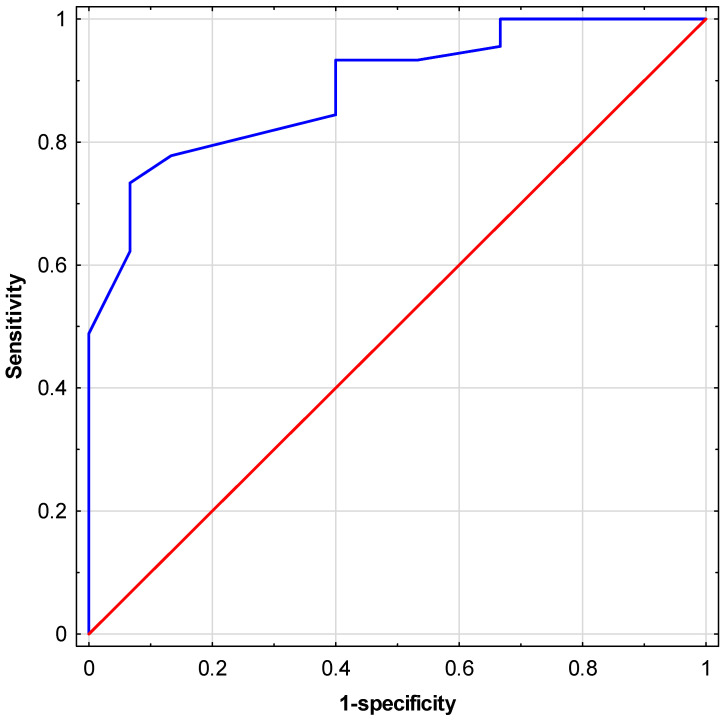
ROC curve for the designed model (blue line, AUC = 0.89). For illustrative purposes, the red line represents a curve where only 50% of outcomes are correctly predicted (AUC = 0.5).

The conformity of fit of the created multivariate logistic regression model was assessed using the Hosmer–Lemeshow goodness-of-fit test, resulting in a high quality of fit (*p* = 0.96). Subsequently, an ROC analysis was conducted, and the area under the curve (AUC) was determined for the predictor created based on the proposed logistic regression model (Figure 8). A high AUC value of 0.89 (95% CI: 0.80; 0.97) was obtained. The OR values obtained in the model for individual independent variables indicate that individuals with insulin resistance diagnosed using the Matsuda index have over 37 times (1/0.0271) lower odds of obtaining semen with correct motility than men without diagnosed insulin resistance. Individuals with fewer than 4 days of sexual abstinence before semen collection had over 20 times (1/0.0477) greater odds of obtaining semen with correct motility than those with 4 days or more of abstinence. On the other hand, patients who consumed vitamin quantities according to established norms showed over 72% greater odds (OR = 1.7243) of obtaining semen with correct motility than men not meeting the required vitamin intake (Table 8).

For the designed model, the cut-off point determined using the minimal sum of squared coordinates was 1.612 (the value of the predictor obtained from the logistic regression equation). For the determined cut-off point, the sensitivity was 77.8%, while the specificity 86.7%.

## 4. Discussion

Sperm motility depends on complex mechanisms, both structural and molecular [1]. In mammalian sperm, the axoneme is covered with additional structures, i.e., a fibrous sheath, a mitochondrial sheath, and dense fibers located at various points of the filament. In humans, the mitochondrial sheath has a spiral shape and wraps around the axoneme, supplying ATP, i.e., the energy necessary for sperm motility. The main component of the axoneme has receptors for signaling proteins that influence motility regulation, as well as proteins involved in hyperactivation and capacitation [52]. These essential structures for semen production are unique to the sperm tail. They provide fertility to semen by offering additional stiffness and the energy necessary for sperm to move within the female reproductive tract—ultimately leading to a higher probability of pregnancy [53]. This shows that although human sperm do not rely solely on mitochondria to produce ATP, the axoneme is an important factor that influences both sperm motility and fertility. At a structural level, this may explain the significance of sperm motility as a key parameter in fertility analyses.

Numerous studies have been performed that investigated the influence of lifestyle components on motility [24,25,26,27,28,54,55], including alcohol consumption [54], tobacco product use [24], obesity [25], sleep deficiency or disorders [26], high consumption of meat products [27], stress [55], and intense physical activity [28]. Researchers have also focused on the significance of the imbalance between antioxidants and free radicals in the testicular microenvironment [56,57], which can result in sperm damage through agglutination, the inhibition of capacitation, reduced motility and cervical mucus penetration ability, or faulty sperm interaction with the egg cell [58]. Hence, an imbalance in the testicular microenvironment is one of the mechanisms linking oxidative stress with motility, and thus also with fertility.

This study showed a positive correlation between VCI, diet quality (DI), and sperm motility. Although the overall correlation between VCI and sperm motility is weak due to a wide variation in sperm motility, when the participants were stratified according to BMI groups, a strong correlation was found between the aforementioned parameters in the obese group, despite the small sample size. This indicates the importance of vitamin supplementation, especially in obese individuals, and could be explained by the fact that obesity may increase the risk of infertility and worsen semen quality by increasing oxidative stress in the semen [59]. Moreover, deficiencies in micronutrients are common among obese individuals, who are particularly susceptible to deficiencies in antioxidants such as vitamin E, vitamin A, vitamin C, folic acid, beta-carotene, selenium, and B vitamins [60]. When investigating supplementation with micronutrients and antioxidant vitamins, McPherson et al. [61] also suggested that fertility issues associated with obesity in men could be reversed through short-term supplementation with micronutrients, particularly if administered during the passage of sperm through the epididymis, when it is the most vulnerable to oxidative damage.

It was also found that men with correct motility scores were characterized by higher mineral consumption levels (MCI) compared to those with incorrect scores. This suggests that a well-balanced diet meeting the requirements for vitamins and mineral components may have a positive impact on fertility. This result is consistent with the study performed by Oostingh et al., which showed a positive correlation between sperm motility and healthy dietary habits [62]. Similarly, Agarwal et al. demonstrated that antioxidant supplementation among men undergoing infertility treatment improves motility [36], while Salas-Huetos et al. showed that the consumption of pro-inflammatory products, the low intake of antioxidants found in food products, and elevated values of the glycemic index and high glycemic load in the diet lead to increased oxidative stress, i.e., increased production of pro-inflammatory cytokines in the body [37]. The effectiveness of supplementation with antioxidant vitamins and minerals in infertility treatment is supported by numerous studies [56,57], while research on male fertility suggests that vitamin C combined with L-carnitine and tocopherol should be used in the diagnosis of oxidative stress in men planning to have children. Another effective combination is supplementation with selenium and N-acetylcysteine, which improves hormonal balance. Moreover, it is beneficial to combine selenium with vitamin E and zinc as this combination has been shown to rebuild sperm damaged by oxidative stress and improve sperm motility and endurance [36]. Some studies also found benefit in vitamin D supplementation in relation to sperm parameters. Following six months of vitamin D supplementation, a notable improvement in both the mean sperm concentration and the progressive sperm motility was observed in infertile males diagnosed with oligoasthenozoospermia. This, however, does not impact pregnancy outcomes [63]. In contrast, a meta-analysis performed by de Ligny et al. raises concerns as to whether antioxidant supplementation should be recommended to improve male fertility. However, the poor reporting of methods of randomization in the studies used for the meta-analysis as well as their other limitations suggest that a larger study on this topic, better prepared and conducted on a larger number of participants, is necessary [64], with some proposals for protocols already in place [65].

Although the exact relationships between insulin resistance and infertility are still an area of active investigation, the underlying biological mechanisms have been proposed by researchers. Sliwowska et al. [66] explain that insulin, primarily known for its role in regulating blood glucose levels, also influences fat and protein metabolism by enhancing amino acid transport into cells and promoting lipid synthesis while reducing fatty acid release. In addition to its metabolic functions, insulin interacts with the hypothalamic–pituitary–gonadal (HPG) axis, affecting the release patterns of gonadotropin-releasing hormone (GnRH) and luteinizing hormone (LH). The researchers point out that studies show that hypo- or hyperinsulinemia can disrupt GnRH/LH pulsatile and surge release patterns, impacting reproductive processes, although the exact mechanisms remain debated due to various experimental factors such as insulin administration methods and models of hypoinsulinemia. Furthermore, it is essential to emphasize the significance of the insulin resistance-induced disruption of glucose metabolism in spermatogenesis as fundamental cellular processes, including motility, heavily depend on it [40].

Due to their associations with obesity and, consequently, with the possibility of insulin resistance, body composition and body weight are often considered in the context of male fertility. In this study, it was found that participants with healthier body compositions were more likely to achieve correct motility scores. Differences in BFM levels were also observed between men with correct and incorrect sperm motility scores, i.e., the proportion of body fat was significantly lower in the former group. This is an unsurprising result as excess adipocytes have the ability to release adipokines and inflammatory mediators, which disrupt cell function and increase inflammation in the body [42]. Additionally, excess adipose tissue impairs heat circulation, contributing to increased testicular temperature and thus potentially worsening the quality of the produced sperm [45].

Hyperglycemia, resulting from an improper diet and often associated with obesity, negatively affects sperm motility due to the fact that glucose metabolism significantly influences spermatogenesis [37]. In order to access the testes, glucose must penetrate the blood–testis barrier. This is possible through facilitated diffusion via glucose transporters (GLUT) and is dependent on the redistribution of GLUT in the cell membrane as well as the overall level of GLUT. Research results indicate that glucose transporter 1 (GLUT1) and glucose transporter 3 (GLUT3) play a synergistic role in maintaining glucose uptake in Sertoli cells to ensure lactate production, and subsequently ATP utilized by sperm for movement [67]. Scientists attempted to link lifestyle and its individual components to various semen parameters, demonstrating that a “sweet diet” positively correlated with sperm motility (*p* = 0.017), which may be associated with obtaining energy from simple sugars [68]. Moreover, studies on fertility problems in diabetes, i.e., the condition associated with the presence of hyperglycemia and insulin resistance, are already well known in the scientific world [22,23,34]; however, the number of studies directly linking sperm quality with insulin resistance is limited.

In this study, it was observed that non-insulin-resistant men were more likely to achieve correct sperm motility scores compared to men with insulin resistance. Additionally, lower fasting insulin levels and higher Matsuda index scores were found in men with correct sperm motility scores compared to those with incorrect motility scores. Results of the study performed by Ma et al. indicate that the HOMA-IR index was an independent factor influencing progressive motile sperm [21]. Similarly, Andlib et al. reported that insulin resistance may impair the function of the hypothalamus, pituitary gland, and gonads, resulting in reduced secretion of reproductive hormones such as GnRH (gonadotropin-releasing hormone), FSH (follicle-stimulating hormone), LH (luteinizing hormone), and testosterone. These findings reflect those obtained for insulin deficiency. Researchers also emphasize that insulin resistance affects glucose metabolism, which is responsible for the motility and fertilizing activity of mature sperm [34]. Based on these findings, it can be concluded that insulin resistance, not necessarily in conjunction with diabetes, affects sperm motility and, consequently, semen quality and male fertility in general.

Some studies suggest shortening the duration of sexual abstinence before semen collection for Assisted Reproduction Techniques (ARTs) to prevent a decline in semen quality. Shorter abstinence significantly improves motility and correlates positively with ART outcomes in couples with infertility issues due to the male factor. Recent research also indicates semen freshness as a possible determinant of a higher number of live births [69]. In contrast, Sauer et al. found that the duration of sexual abstinence did not affect sperm motility, but it is worth noting that the maximum duration of sexual abstinence in this study was only 120 h [70]. Overall, most studies suggest that the duration of sexual abstinence does affect sperm motility, with the ideal duration that would lead to obtaining the highest values of this parameter of up to 4 days [46,47,48]. This conclusion was confirmed in the present study that 83% of men reporting sexual abstinence of fewer than 4 days achieved positive sperm motility results, compared to those reporting sexual abstinence of 4 days or longer (only 50%). In itself, sexual abstinence does not constitute a parameter strictly related to lifestyle; however, it would seem necessary to use the findings discussed above to establish standards in sample collection for fertility tests. Nonetheless, the duration of sexual abstinence appears to have a significant impact on semen quality in men, particularly as far as motility is concerned [69]. This relationship suggests that making clinical decisions based on data from samples collected at an inappropriate time may prove to be incorrect.

As part of this study, a multivariate logistic regression model was created, demonstrating the simultaneous influence of three parameters, i.e., the Matsuda index, VCI, and the duration of sexual abstinence, on motility scores. The model indicates that the presence of insulin resistance and an excessively long duration of sexual abstinence will negatively impact sperm motility, while adequate vitamin intake will have a positive effect on the parameter. The very high area under the ROC curve obtained for the predictor developed based on the model indicates its high effectiveness in predicting correct or incorrect sperm motility scores. It is also worth noting that each of the parameters included in the model has a different nature, which results in various diagnostic and clinical implications. Considering the above, the multivariate model can be considered an effective and practical tool to be utilized in infertility research, especially in the context of the presence or absence of insulin resistance.

The inclusion of insulin resistance in the model confirms the association between this condition and male fertility. However, as the condition is modifiable by lifestyle factors to a minimal degree, its presence or absence should primarily be regarded as a clinical feature that increases the likelihood of fertility problems in the studied men. It would also be interesting to relate the prevalence of insulin resistance in today’s society and its status as a civilization disease to the similar features of infertility, i.e., its growing incidence and civilization-related development. The ever-lowering norms for parameters describing fertility also signify that the progress of civilization constitutes an overreaching cause of the two issues [71]. In this context, the inclusion of insulin resistance in the logistic regression model developed in this study may be interpreted as a reflection of the connections between various civilization diseases and their consequences.

The presence of vitamin supplementation in the model has the greatest clinical implications. This is due to the fact that the factor is easily modifiable, with adequate doses of vitamins possibly exhibiting a tangible effect on improving semen parameters, and thus male fertility. The antioxidant potential of vitamins has protective effects on both sperm DNA and motility. Vitamin C has the ability to neutralize damage caused by free radicals in semen, thus increasing motility, and reducing DNA fragmentation and lipid peroxidation levels [72]. Vitamin E, found in vegetable oils, has the ability to repair oxidative radicals—preventing increased lipid peroxidation and mitigating the negative effects of oxidative stress on male fertility [73]. In addition to antioxidant vitamins, an adequate intake of B-group vitamins and vitamin D may also play a significant role in male fertility. Vitamin B12 enhances the functionality of reproductive organs, reduces the production of free radicals and homocysteine, which are toxic to sperm DNA, and decreases inflammation [74]. Both reduced and elevated serum levels of vitamin D increase the incidence of abnormalities in sperm morphology and negatively affect progressive motility and total sperm count [75]. It should be emphasized that although various modifiable parameters impact fertility, a multivariate model (characterized by a high AUC) that would include insulin resistance could only be successfully constructed for vitamins.

Salas-Huetos et al. created six different multidimensional linear regression models to assess the relationship between adherence to the Mediterranean diet and semen parameters. The data were adjusted for age, energy intake, and BMI. It was shown that men who adhered to the Mediterranean diet rigorously exhibited total sperm motility scores compared to those adhering to the Mediterranean diet to a lesser extent [76]. Although the researchers focused on adherence to a specific diet, knowledge concerning the high content of antioxidant vitamins in the Mediterranean diet makes it possible to conclude that these results are consistent with the implications of the multivariate model designed in this study.

## 5. Conclusions

The results of this study point to the existence of an association between certain lifestyle factors and semen quality, as measured using sperm motility as the key indicator. Additionally, a significant relationship between the presence or absence of insulin resistance and male fertility has been demonstrated. Most importantly, this study led to the construction of a multivariate model which makes it possible to predict the outcome regarding correct or incorrect motility scores on the basis of three independent parameters, i.e., the Matsuda index, VCI, and the duration of sexual abstinence.

In general, the lifestyle choices and habits typical for modern civilization often have a negative impact on male fertility [6]. This can be counteracted by implementing lifestyle modifications, including dietary changes, which may ultimately halt or even reverse this unfavorable trend. The fact that the norms regarding semen quality are being systematically reduced further confirms the existence of a deepening trend of declining semen quality. Coupled with a lifestyle increasingly reliant on the achievements of civilization, the process will remain an increasingly concerning global issue for the foreseeable future.

The study results indicate that the influence of lifestyle factors on male fertility is of a key importance, particularly in the context of insulin resistance, whose presence or absence is an indicator of potential fertility issues. What this finding signifies is that diagnostics towards insulin resistance should be routinely included in fertility-related interventions and procedures.

Modifiable lifestyle factors that influence fertility primarily include vitamin and mineral consumption, in connection with dietary modifications in line with WHO recommendations, with a particular emphasis on diets containing large amounts of antioxidant-rich foods, especially the Mediterranean diet. The results of this study show that vitamin intake levels correlate with motility scores. In addition, the presented multivariate model indicates the considerable influence of vitamin intake on fertility. The results of this study show that the issue of adequate micronutrient supplementation targeting fertility outcomes requires further research, particularly studies that would focus on designing vitamin consumption regimens tailored to the needs of men who want to improve their fertility scores.

As far as the association between the duration of sexual abstinence and semen quality is concerned, the reduced motility after the fourth day of abstinence should be considered in the process of semen collection for diagnostic purposes in order to avoid false-negative results and erroneous clinical decisions.

In view of the findings of this study, research on the impact of insulin resistance and modifiable lifestyle factors appears to be a promising direction as far as semen quality studies are concerned. Moreover, to validate the strength of the proposed multivariate model, further prospective studies should be conducted using new, independent, and larger datasets—based on introducing lifestyle modifications—to assess whether increasing micronutrient (particularly vitamin) intake to the adequate levels and improving selected lifestyle factors in men with insulin resistance could enhance their fertility. It would also be beneficial to verify the existence of a correlation between the Matsuda index and sperm motility in a larger group of patients. This will be evaluated in the authors’ next planned study, where additional parameters will be examined, particularly concerning carbohydrate metabolism disorders and the determination of vitamin levels in the blood.

## 6. Limitations of This Study

The results of this study could be more robust if the study group was larger, especially due to the relatively small effect size of the tested phenomena. In addition, the clinical–control characteristic of this study makes its results more difficult to interpret than would be the case in a prospective study. For this reason, the authors intend to follow up these findings with a larger prospective study. Due to the fact that this study was coordinated and performed in two units, some patients failed to complete the entire planned set of tests, which resulted in a reduction in the size of the study group. Additionally, since the topic of semen donation and quality is an intimate matter in certain environments, this limits the number of people willing to participate in a study focused on such matters. Another issue connected with the design of this study is recall bias inherent in the use of the three-day food diary. This was mitigated by the researchers emphasizing the importance of the accurate recall and recording of data when instructing the participants on how to complete the diary.

## Figures and Tables

**Table 1:** The two cohorts of subjects included in this study (with and without insulin resistance).

Parameter	*n*	Insulin Resistance Median (Q_1_; Q_3_)	*n*	No insulin Resistance Median (Q_1_; Q_3_)
Age	48	28.00 (25.00; 32.00)	28	26.50 (25.00; 30.00)
BMI	48	28.96 (26.74; 33.05)	28	24.41 (22.28; 27.84)
Physical activity (hours/week)	40	0.50 (0.50; 3.00)	19	3.00 (0.50; 3.00)
TBW (Total Body Water)	48	50.55 (47.20; 55.15)	28	49.95 (45.75; 54.75)
BFM (Body Fat Mass)	48	26.70 (21.00; 36.75)	28	14.15 (10.60; 17.50)
SMI (Skeletal Muscle Index)	48	41.34 (38.14; 44.02)	28	47.65 (43.71; 49.34)
Sperm concentration per mL	45	29.48 (14.37; 46.88)	28	30.66 (12.45; 54.04)
Sperm concentration per ejaculate	45	69.53 (40.71; 113.82)	28	101.51 (26.53; 181.96)
Progressive sperm motility	45	34.78 (19.04; 47.64)	28	37.45 (29.59; 46.85)
Sperm motility	45	48.64 (34.69; 69.02)	28	58.18 (46.83; 70.94)
Sperm morphology	45	8.00 (3.00; 13.00)	28	6.00 (2.50; 9.50)
Fasting insulin	48	8.90 (7.10; 12.45)	28	4.00 (2.80; 5.55)
Cholesterol level	48	179.50 (163.50; 204.00)	28	170.00 (151.00; 182.00)
LDL cholesterol level	48	122.00 (103.50; 145.00)	28	105.50 (84.50; 127.50)
HDL cholesterol level	48	50.00 (39.00; 56.00)	28	52.50 (44.50; 63.00)
Triglyceride level	48	117.00 (83.50; 141.50)	28	75.00 (46.50; 92.50)

**Table 2:** Results for different motility parameters in the two cohorts of subjects (with and without insulin resistance).

Parameter	*n*	Insulin Resistance Median (Q_1_; Q_3_)	*n*	No Insulin Resistance Median (Q_1_; Q_3_)
Fast progressive motility [%]	45	19.86 (8.89; 31.13)	28	20.13 (9.15; 32.70)
Slow progressive motility [%]	45	13.63 (10.18; 11.80)	28	15.68 (10.71; 19.12)
Non-progressive motility [%]	45	13.86 (10.66; 17.86)	28	16.58 (12.77; 23.71)
Immobile sperm [%]	45	47.18 (30.98; 58.22)	28	38.02 (28.65; 50.65)
Hyperactive sperm [%]	45	0.00 (0.00; 0.00)	28	0.00 (0.00; 0.00)
Mucus penetration [%]	45	33.87 (22.03; 43.78)	28	34.46 (16.95; 43.01)

**Table 3:** Relationship between body composition of the participants and sperm motility.

	Body Composition Index (BCI) [0–3]					
Motility	0	1	2	3	Total	*p*-Value
Incorrect	8 (50%)	6 (25%)	3 (14.29%)	1 (8.33%)	18	0.04
Correct	8 (50%)	18 (75%)	18 (85.71%)	11 (91.67%)	55	
Total	16	24	21	12	73	

**Table 4:** Relationship between the presence of insulin resistance as measured using the HOMA-IR index and sperm motility.

	HOMA-IR IR Diagnosis			
Motility	No Insulin Resistance	Insulin Resistance	Total	*p*-Value
Incorrect	8 (17.02%)	10 (38.46%)	18	0.04
Correct	39 (82.98%)	16 (61.54%)	55	
Total	47	26	73	

**Table 5:** Relationship between the presence of insulin resistance measured using the Matsuda index and sperm motility.

	Matsuda Index IR Diagnosis			
Motility	No Insulin Resistance	Insulin Resistance	Total	*p*-Value
Incorrect	3 (10.71%)	15 (33.33%)	18	0.03
Correct	25 (89.29%)	30 (66.67%)	55	
Total	28	45	73	

**Table 6:** Relationship between the duration of sexual abstinence declared by the participants and sperm motility.

	Sexual Abstinence			
Motility	Less than 4 Days	4 Days and More	Total	*p*-Value
Incorrect	8 (16.67%)	9 (50%)	17	0.006
Correct	40 (83.33%)	9 (50%)	49	
Total	48	18	66	

**Table 7:** Univariate logistic regression analysis in relation to sperm motility scores.

Variable	Odds Ratio	95% Confidence Interval		*p*-Value
Matsuda index	0.2400	0.0623	0.9243	0.04
HOMA-IR index	0.3282	0.1096	0.9828	<0.05
MacCI	0.5589	0.3017	1.0353	0.06
MCI	1.6691	1.0633	2.6200	0.03
Tobacco smoking	1.8667	0.4638	7.5131	0.38
Physical activity	0.3958	0.1156	1.3552	0.14
BMI	0.5133	0.2438	1.0811	0.08
Age	0.9904	0.9152	1.0718	0.81
VCI	1.5094	1.1087	2.0551	0.009
Duration of sexual abstinence	0.2000	0.0605	0.6612	0.008

**Table 8:** Multivariate logistic regression model in relation to sperm motility scores.

Variable	Odds Ratio	95% Confidence Interval		*p*-Value
Matsuda index	0.0271	0.0016	0.4610	0.01
VCI	1.7243	1.0680	2.7837	0.03
Duration of sexual abstinence	0.0477	0.0059	0.3863	0.004

## Data Availability

The data presented in this study are available on request from the corresponding author.
